# Morpho-molecular diversity of Linocarpaceae (Chaetosphaeriales): *Claviformispora* gen. nov. from decaying branches of *Phyllostachys heteroclada*

**DOI:** 10.3897/mycokeys.70.54231

**Published:** 2020-07-16

**Authors:** Xiu-Lan Xu, Chun-Lin Yang, Rajesh Jeewon, Dhanushka N. Wanasinghe, Ying-Gao Liu, Qian-Gang Xiao

**Affiliations:** 1 Research Institute of Forestry, Chengdu Academy of Agricultural and Forestry Sciences, Nongke Road 200, Chengdu 611130, China; 2 National Forestry and Grassland Administration Key Laboratory of Forest Resources Conservation and Ecological Safety on the Upper Reaches of the Yangtze River, Sichuan Agricultural University, Wenjiang District, Huiming Road 211, Chengdu 611130, China; 3 Sichuan Province Key Laboratory of Ecological Forestry Engineering on the Upper Reaches of the Yangtze River, Sichuan Agricultural University, Wenjiang District, Huiming Road 211, Chengdu 611130, China; 4 Department of Health Sciences, Faculty of Science, University of Mauritius, Reduit, Mauritius; 5 Key Laboratory for Plant Diversity and Biogeography of East Asia, Kunming Institute of Botany, Chinese Academy of Science, Kunming 650201, Yunnan, China

**Keywords:** bambusicolous fungi, one new genus and species, phylogeny, taxonomy

## Abstract

In this paper, *Claviformispora***gen. nov.** in Linocarpaceae is introduced from *Phyllostachys heteroclada* in Sichuan Province, China. The new genus is characterised by its distinct morphological characters, such as ostiole with periphyses, asci with a thick doughnut-shaped, J- apical ring and clavate ascospore without septum-like band and appendage. Maximum Likelihood and Bayesian Inference phylogenetic analyses, based on DNA sequence data from ITS, LSU, SSU and TEF-1α regions, provide further evidence that the fungus is a distinct genus within this family. The new genus is compared with similar genera, such as *Linocarpon* and *Neolinocarpon*. Descriptions, illustrations and notes are provided for the new taxon.

## Introduction

The order Chaetosphaeriales Huhndorf, A.N. Mill. & F.A. Fernández (Sordariomycetes) was introduced in Sordariomycetidae O.E. Erikss. & Winka, based on LSU sequence data (Huhndorf et al. 2004) and currently comprises four families viz. Chaetosphaeriaceae Réblová, M.E. Barr & Samuels, Helminthosphaeriaceae Samuels, Cand. & Magni, Leptosporellaceae S. Konta & K.D. Hyde and Linocarpaceae S. Konta & K.D. Hyde (Hongsanan et al. 2017; Konta et al. 2017; Wijayawardene et al. 2020). Recently, 43 genera were accepted within Chaetosphaeriaceae and seven genera within Helminthosphaeriaceae (Hyde et al. 2020; Wijayawardene et al. 2020). Based on morphology and combined analyses of ITS and LSU sequence data, Konta et al. (2017) accommodated *Linocarpon* Syd. & P. Syd. and *Neolinocarpon* K.D. Hyde in Linocarpaceae and *Leptosporella* Penz. & Sacc. in Leptosporellaceae. Leptosporellaceae and Linocarpaceae are morphologically similar in their dome-shaped ascomata and filiform ascospores (Hyde and Alias 1999; Cai et al. 2004; Konta et al. 2017). The former, however, can be delineated based on ascospores that are narrow, long filiform, with gradually tapering ends and indistinct mucilage (if present), whereas in Linocarpaceae, ascospores have a distinct appendage at the apex and are generally wider and differ in appearance at the ends (Konta et al. 2017).

The genus *Leptosporella* was introduced with *L. gregaria* Penz. & Sacc., 1897 as the type species by Penzig and Saccardo (1897). Lumbsch and Huhndorf (2010) referred it in Sordariomycetidae genera *incertae sedis*. Subsequently, the genus was referred to the Chaetosphaeriales, based on phylogenetic analysis of LSU sequence data (Huhndorf and Miller 2011; Dai et al. 2016; Wijayawardene et al. 2020). At present, 15 epithets of *Leptosporella* are recorded in Index Fungorum (http://www.speciesfungorum.org/Names/Names.asp). Sydow and Sydow (1917) introduced *Linocarpon* with *L. pandani* Syd. & P. Syd., 1917 as the type species. Hyde (1992a) introduced *Neolinocarpon* to accommodate a linocarpon-like species, *N. globosicarpum* K.D. Hyde, 1992. Currently in Index Fungorum (2020), 44 and 13 epithets are accommodated in *Linocarpon* and *Neolinocarpon*, respectively. Hyde (1997) and Jeewon et al. (2003) reported that *Linocarpon* and *Neolinocarpon* can be accommodated in Hyponectriaceae (Xylariales), while Bahl (2006) suggested a closer relationship with Chaetosphaeriales and Xylariales, based on their molecular data. However, with more taxon sampling and fresh collections, Konta et al. (2017) confirmed that *Linocarpon* and *Neolinocarpon* should be accommodated in a distinct family (Linocarpaceae) in Chaetosphaeriales.

The present research is a part of our investigations on the taxonomic and phylogenetic circumscriptions of pathogenic and saprobic micro-fungi associated with bamboo in Sichuan Province, China. In this paper, we introduce a new genus *Claviformispora* in Linocarpaceae, typified by *C. phyllostachydis* from *Phyllostachys heteroclada* Oliv., 1894 (Poaceae). The morphological differences and analyses of a combined ITS, LSU, SSU and TEF-1α sequence dataset support the validity of the new genus and its placement in Linocarpaceae. The new genus is compared with other genera in the family. The comprehensive descriptions and micrographs of new taxa are provided.

## Materials and methods

### Specimen collection and morphological study

Bamboo materials were collected from Ya’an City, China. Single ascospore isolations were carried out following the method described by Chomnunti et al. (2014) and the germinating spores were transferred to PDA, incubated at 25 °C in the dark and cultural characteristics were determined. Ascomata were observed and photographed using a dissecting microscope NVT-GG (Shanghai Advanced Photoelectric Technology Co. Ltd, China) matched to a VS-800C micro-digital camera (Shenzhen Weishen Times Technology Co. Ltd., China). The anatomical details were visualised using Nikon ECLIPSE *Ni* compound microscope fitted to a Canon 600D digital camera and an OPTEC BK-DM320 microscope matched to a VS-800C micro-digital camera (Shenzhen Weishen Times Technology Co. Ltd., China). Iodine reaction of the ascus wall was tested in Melzer’s reagent (MLZ). Lactate cotton blue reagent was used to observe the number of septa. The gelatinous appendage was observed in Black Indian ink. Type specimens were deposited at the Herbarium of Sichuan Agricultural University, Chengdu, China (SICAU) and Mae Fah Luang University Herbarium (MFLU). The ex-type living cultures are deposited at the Culture Collection in Sichuan Agricultural University (SICAUCC) and the Culture Collection at Mae Fah Luang University (MFLUCC). Index Fungorum numbers (http://www.indexfungorum.org/Names/Names.asp) are registered and provided.

### DNA extraction, PCR amplification and DNA sequencing

Total genomic DNA was extracted from mycelium that were grown on PDA at 25 °C for two weeks using a Plant Genomic DNA extraction kit (Tiangen, China) following the manufacturer’s instructions. The primers pairs LR0R and LR5 (Vilgalys and Hester 1990), NS1 and NS4, ITS5 and ITS4 (White et al. 1990), EF1-983F and EF1-2218R (Rehner 2001) were used for the amplification of the partial large subunit nuclear rDNA (LSU), the partial small subunit nuclear rDNA (SSU), internal transcribed spacers (ITS) and translation elongation factor 1-alpha (TEF-1α), respectively.

Polymerase chain reaction (PCR) was performed in 25 μl final volumes containing 22 μl of Master Mix (Beijing TsingKe Biotech Co. Ltd.), 1 μl of DNA template, 1 μl of each forward and reverse primers (10 μM). The PCR thermal cycle programmes for LSU, SSU, ITS and TEF1-α gene were amplified as: initial denaturation 94 °C for 3 minutes, followed by 35 cycles of denaturation at 94 °C for 30 seconds, annealing at 55 °C for 50 seconds, elongation at 72 °C for 1 minute and final extension at 72 °C for 10 minutes. PCR products were sequenced with the above-mentioned primers at TsingKe Biological Technology Co. Ltd, Chengdu, China. The newly-generated sequences from the LSU, SSU, TEF-1α and ITS regions were deposited in GenBank (Table 1).

### Phylogenetic analyses

Taxa to be used for phylogenetic analyses were selected, based on results generated from nucleotide BLAST searches online in GenBank and recent publications (Lu et al. 2016; Konta et al. 2017; Senwanna et al. 2018; Wei et al. 2018; Lin et al. 2019). *Gelasinospora tetrasperma* (CBS 178.33) and *Sordaria fimicola* (CBS 508.50) were selected as the outgroup taxa. The sequences were downloaded from GenBank (http://www.ncbi.nlm.nih.gov/) and the accession numbers are listed in Table 1. A combined ITS, LSU, SSU and TEF-1α sequence dataset was used to construct the phylogenetic tree. DNA alignments were performed by using MAFFT v.7.429 online service (Katoh et al. 2019) and ambiguous regions were excluded with BioEdit version 7.0.5.3 (Hall 1999). Multigene sequences were concatenated by using Mesquite software (Maddison and Maddison 2019). Maximum Likelihood (ML) and Bayesian Inference (BI) analyses were performed. The best nucleotide substitution model was determined by MrModeltest v. 2.2 (Nylander 2004).

Maximum Likelihood analysis and Bayesian Inference analysis were generated by using the CIPRES Science Gateway web server (Miller 2010). RAxML-HPC2 on XSEDE (8.2.10) (Stamatakis 2014) with GTR+GAMMA substitution model with 1000 bootstrap iterations was chosen for Maximum Likelihood analysis. For BI analyses, the best-fit model GTR+I+G for ITS, LSU and SSU was selected in MrModeltest 2.2 and GTR+G for TEF. The analyses were computed with six simultaneous Markov Chain Monte Carlo (MCMC) Chains with 8,000,000 generations and a sampling frequency of 100 generations. The burn-in fraction was set to 0.25 and the run automatically ended when the average standard deviation of split frequencies reached below 0.01.

Phylogenetic trees were visualised with FigTree v.1.4.3 (Rambaut and Drummond 2016) and edited using Adobe Illustrator CS6 (Adobe Systems Inc., United States). Maximum Likelihood bootstrap values (MLBP) equal to or greater than 70% and Bayesian Posterior Probabilities (BYPP) equal to or greater than 0.95 were accepted. The finalised alignment and tree were deposited in TreeBASE (http://www.treebase.org), submission ID: 25996. The new taxa introduced follow the recommendations of Jeewon and Hyde (2016).

## Results

### Phylogenetic analyses

**Figure 1.**
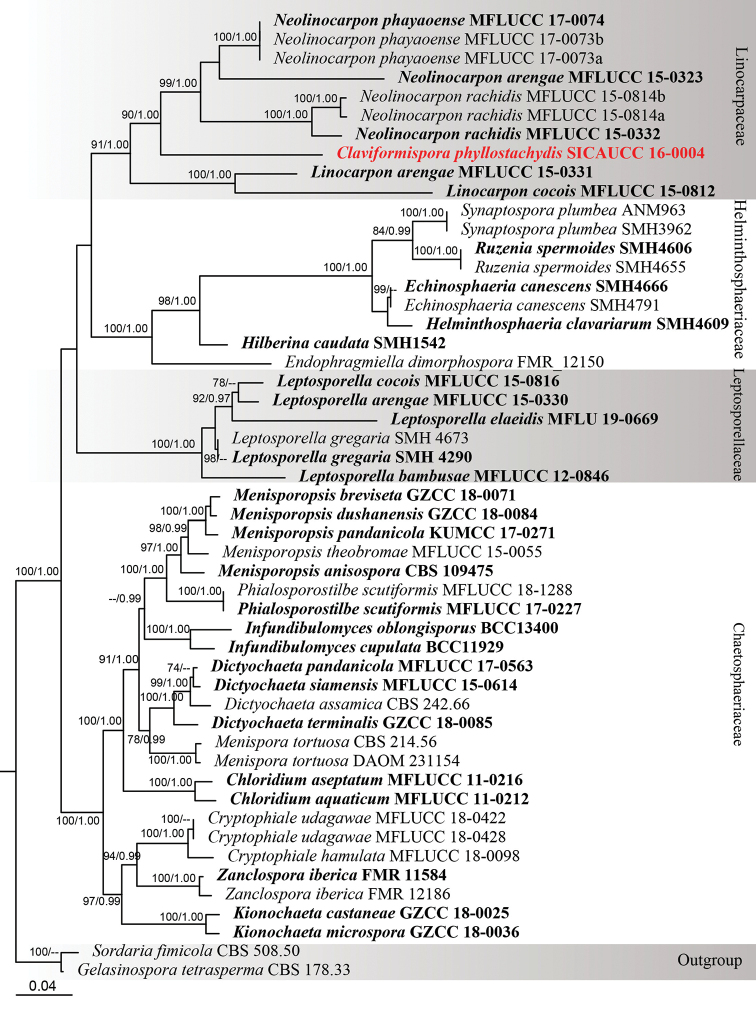
Phylogram of RAxML analysis based on a combined ITS, LSU, SSU and TEF-1α sequence dataset within order Chaetosphaeriales. Bootstrap support values for maximum likelihood (ML, left) greater than 70% and Bayesian posterior probabilities (PP, right) equal to or greater than 0.95 are indicated at the nodes. The tree is rooted to *Gelasinospora tetrasperma* (CBS 178.33) and *Sordaria fimicola* (CBS 508.50). All sequences from ex-type strains are in bold. The newly-generated sequence is in red.

Phylogenetic analyses of a combined dataset (ITS, LSU, SSU, TEF-1α) comprises 51 taxa within the order Chaetosphaeriales (Table 1), including 24 taxa in family Chaetosphaeriaceae, nine taxa in Helminthosphaeriaceae, ten taxa in Linocarpaceae, six taxa in Leptosporellaceae and two outgroup taxa in Sordariales. The dataset consisted of 5,849 characters including gaps (LSU = 1,571, ITS = 736, SSU = 2,522, TEF = 1,020). The best scoring tree of RAxML analysis is shown in Fig. 1, with the support values of ML and BI analyses.

**Table 1.** Molecular data used in this study and GenBank accession numbers.

Species name	Strain	GenBank accession number			
		LSU	ITS	SSU	TEF
*Chloridium aquaticum*	MFLUCC 11-0212	MH476567	MH476570	MH476573	–
*Chloridium aseptatum*	MFLUCC 11-0216	MH476568	NR_158365	MH476574	–
***Claviformispora phyllostachydis***	**SICAUCC 16-0004**	**MT232720**	**MT232736**	**MT232735**	**MT240855**
*Cryptophiale hamulata*	MFLUCC 18-0098	MG386756	–	MG386757	–
*Cryptophiale udagawae*	MFLUCC 18-0422	MH758211	MH758198	MH758205	–
	MFLUCC 18-0428	MH758210	MH758197	MH758204	–
*Dictyochaeta siamensis*	MFLUCC 15-0614	KX609952	KX609955	–	–
*Dictyochaeta assamica*	CBS 242.66	MH870426	MH858788	–	–
*Dictyochaeta pandanicola*	MFLUCC 17-0563	MH376710	MH388338	MH388307	MH388373
*Dictyochaeta terminalis*	GZCC 18-0085	MN104624	MN104613	MN104633	–
*Echinosphaeria canescens*	SMH4666	KF765605	–	–	–
	SMH4791	AY436403	–	–	–
*Endophragmiella dimorphospora*	FMR_12150	KY853502	KY853442	HF937351	–
*Gelasinospora tetrasperma*	CBS 178.33	DQ470980	NR_077163	DQ471032	DQ471103
*Helminthosphaeria clavariarum*	SMH4609	AY346283	–	–	–
*Hilberina caudata*	SMH1542	KF765615	–	–	–
*Infundibulomyces cupulata*	BCC11929	EF113979	EF113976	EF113982	–
*Infundibulomyces oblongisporus*	BCC13400	EF113980	EF113977	EF113983	–
*Kionochaeta castaneae*	GZCC 18-0025	MN104621	MN104610	MN104630	–
*Kionochaeta microspora*	GZCC 18-0036	MN104618	MN104607	MN104627	–
*Leptosporella arengae*	MFLUCC 15-0330	MG272246	MG272255	MG366594	MG272259
*Leptosporella bambusae*	MFLUCC 12-0846	KU863122	KU940134	–	–
*Leptosporella cocois*	MFLUCC 15-0816	–	MG272256	–	–
*Leptosporella gregaria*	SMH4290	AY346290	–	–	–
	SMH4673	HM171287	–	–	–
*Leptosporella elaeidis*	MFLU 19-0669	MK659772	MK659767	MK659774	MN883560
*Linocarpon arengae*	MFLUCC 15-0331	MG272247	–	MG366596	–
*Linocarpon cocois*	MFLUCC 15-0812	MG272248	MG272257	MG272253	–
*Menispora tortuosa*	DAOM 231154	AY544682	KT225527	AY544723	–
	CBS 214.56	AF178558	AF178558	–	–
*Menisporopsis anisospora*	CBS 109475	MH874421	MH862827	–	–
*Menisporopsis breviseta*	GZCC 18-0071	MN104623	MN104612	MN104632	–
*Menisporopsis dushanensis*	GZCC 18-0084	MN104626	MN104615	MN104635	–
*Menisporopsis pandanicola*	KUMCC 17-0271	MH376726	MH388353	MH388320	MH388388
*Menisporopsis theobromae*	MFLUCC 15-0055	KX609954	KX609957	–	–
*Neolinocarpon arengae*	MFLUCC 15-0323	MG272249	MG272258	MG366597	–
*Neolinocarpon rachidis*	MFLUCC 15-0332	MG272250	–	MG366598	–
	MFLUCC 15-0814a	MK106353	MK106342	MK106367	–
	MFLUCC 15-0814b	MK106354	–	MK106368	
*Neolinocarpon phayaoense*	MFLUCC 17-0073a	MG581933	–	MG581936	MG739512
	MFLUCC 17-0073b	MG581934	–	MG581937	MG739513
	MFLUCC 17-0074	MG581935	–	MG581938	MG739514
*Phialosporostilbe scutiformis*	MFLUCC 17-0227	MH758207	MH758194	MH758201	–
	MFLUCC 18-1288	MH758212	MH758199	–	–
*Ruzenia spermoides*	SMH4606	AY436422	–	–	–
	SMH4655	KF765619	–	–	–
*Synaptospora plumbea*	ANM963	KF765620	–	–	–
	SMH3962	KF765621	–	–	–
*Sordaria fimicola*	CBS 508.50	MH868251	MH856730	–	–
*Zanclospora iberica*	FMR 11584	KY853544	KY853480	HF937360	–
	FMR 12186	KY853545	KY853481	HF937361	–

**Notes.** New species in this study is in bold. “–” means that the sequence is missing or unavailable. **Abbreviations. ANM**: Collection of A.N. Miller; **BCC**: BIOTEC Culture Collection, National Center for Genetic Engineering and Biotechnology (BIOTEC), Bangkok, Thailand; **CBS**: Centraalbureau voor Schimmelcultures, Utrecht, Netherlands; **DAOM**: Canadian Collection of Fungal Cultures, Agriculture and Agri-Food Canada, Ottawa, Canada; **FMR**: Facultad de Medicina, Universitat Rovira i Virgili, Reus, Tarragona, Spain; **GZCC**: Guizhou Culture Collection, Guiyang, China; **KUMCC**: Kunming Institute of Botany Culture Collection, Chinese Academy of Sciences, Kunming, China; **MFLU**: Herbarium of Mae Fah Luang University, Chiang Rai, Thailand; **MFLUCC**: Mae Fah Luang University Culture Collection, Chiang Rai, Thailand; **SICAUCC**: Sichuan Agricultural University Culture Collection, Sichuan, China; **SMH**: Collection of S.M. Huhndorf.

The best scoring RAxML tree with the final optimisation had a likelihood value of -26,415.700648. The matrix had 1,751 distinct alignment patterns and 64.64% in this alignment is the gaps and completely undetermined characters. Estimated base frequencies were as follows: A = 0.236065, C = 0.261532, G = 0.295313, T = 0.207091, with substitution rates AC = 1.062535, AG = 1.855434, AT = 0.940219, CG = 1.052604, CT = 4.590285, GT = 1.000000. The gamma distribution shape parameter α = 0.311923 and the Tree-Length = 2.281738. The Bayesian analysis resulted in 20,502 trees after 8,000,000 generations. The first 25% of trees (1,624 trees), which represent the burn-in phase of the analyses were discarded, while the remaining 4,878 trees were used for calculating posterior probabilities. Bayesian posterior probabilities were evaluated by MCMC with a final average standard deviation of split frequencies = 0.009877.

Phylogenetic trees generated from Maximum Likelihood (ML) and Bayesian Inference analyses were similar in overall topologies. Phylogeny from the combined sequence data analysis indicates that all families were monophyletic with strong bootstrap support values (Fig. 1). Phylogenetic results show that our novel species *Claviformispora phyllostachydis* (SICAUCC 16-0004) belongs to family Linocarpaceae with 91% ML and 1.00 BYPP support and close to genera *Neolinocarpon* and *Linocarpon* (Fig. 1). The new genus *Claviformispora* constituted a distinct lineage in the family Linocarpaceae (Fig. 1).

## Taxonomy

### Linocarpaceae

Taxon classificationFungiChaetosphaerialesLinocarpaceae

S. Konta & K.D. Hyde, Mycosphere 8(10): 1962 (2017) emend.

EAC8D5F6-CFA5-58FF-AF94-4E44E9D26F1F

#### Type genus.

*Linocarpon* Syd. & P. Syd.

#### Description.

*Saprobic* and *endophytic* fungi on monocotyledons and rarely dicotyledons. ***Sexual morph***: *Ascomata* solitary or aggregated, superficial or immersed comprising black, dome-shaped or subglobose, slightly raised blistering areas with a central ostiole or immersed with a black shiny papilla. *Peridium* composed of dark brown to black cells of *textura angularis*. *Hamathecium* comprising septate paraphyses that are longer than asci, wider at the base, tapering towards the apex. *Asci* 8-spored, unitunicate, cylindrical, with a J-, apical ring, developing from the base and periphery of the ascomata. *Ascospores* parallel or spiral in asci, hyaline or pale yellowish in mass, filiform or claviform, straight or curved, unicellular with or without refringent bands, with or without polar appendages. ***Asexual morph***: *Phialophora*-like spp. were found in *Linocarpon appendiculatum* and *L. elaeidis* cultures (Hyde 1992b), but no records are available for other species.

#### Notes.

Linocarpaceae was introduced as a new family to accommodate *Linocarpon* and *Neolinocarpon* species, based on morphology and phylogeny (Konta et al. 2017). Appressoria were first recorded from *Neolinocarpon rachidis* (Hyde et al. 2019). The new genus *Claviformispora*, which is well-supported within Linocarpaceae suggests that there is a need to amend the morphological circumscriptions of the family given that the ascomata (subglobose) and ascospore (claviform) characters are so different from the other two genera.

### Claviformispora

Taxon classificationFungiChaetosphaerialesLinocarpaceae

X. L. Xu & C. L. Yang gen. nov.

AFA50764-3192-5A3C-8B17-6E9524AE7C3E

Index Fungorum identifier: IF557395

#### Type species.

*Claviformispora phyllostachydis* X. L. Xu & C. L. Yang

#### Etymology.

Name reﬂects the claviform ascospores.

#### Description.

*Saprobic* on dead branches. ***Sexual morph***: *Stromata* solitary or gregarious, black, erumpent. *Ascomata* solitary or aggregated, immersed, subglobose, slightly raised blistering areas with a central ostiole with periphyses. *Peridium* outer cells merging with the host tissues, composed of pale to dark brown cells of *textura angularis*. *Hamathecium* comprising hyaline, septate paraphyses, longer than asci, wider at the base, tapering towards the apex. *Asci* 8-spored, cylindrical to cylindric-clavate, unitunicate, short pedicellate, apically rounded, with a doughnut-shaped, refractive, J- apical ring. *Ascospores* overlapping uniseriate or 2-seriate, clavated with a thin pedicellate, 1-celled, hyaline, without appendage and refringent bands, smooth-walled. ***Asexual morph***: Undetermined.

#### Notes.

*Claviformispora* resembles *Neolinocarpon* in having immersed ascomata and ostiole with periphyses, but differs in forming aggregated ascomata, cylindric-clavate, short pedicellate asci, clavate ascospores with thin pedicel and without septa-like bands and appendages, whereas the ascospores of *Neolinocarpon* and *Linocarpon* (Linocarpaceae) species are usually filiform with refringent bands and appendages (Hyde 1992b, 1997; Konta et al. 2017). The nature of the ascospore appendages appears to be phylogenetically significant for intergeneric delineation as has been seen in other studies (Poonyth et al. 2000; Jeewon et al; 2003, Thongkantha et al. 2003; Cai et al. 2004; Konta et al. 2017), but this warrants further investigations with more sampling and fresh collections of *Neolinocarpon* and *Linocarpon*. Differences in morphology between these genera in Linocarpaceae are summarised in Table 2.

**Table 2.** Morphological comparison of *Linocarpon*, *Neolinocarpon* and *Claviformispora*.

**Morphology**	*** Linocarpon ***	*** Neolinocarpon ***	*** Claviformispora ***
	**(Type: *L. pandani*)**	**(Type: *N. globosicarpum*)**	**(Type: *C. phyllostachydis*)**
Stromata	Absent	Absent	Solitary or aggregated, comprising elliptical areas and large black areas, with slit-like openings
Ascomata	Solitary, superficial, subglobose, comprising black, dome-shaped, raised blistering areas, central ostiole	Solitary, deeply immersed, oval to globose, with central raised, dark, shiny papilla, central ostiole with periphyses	Solitary or aggregated, deeply immersed, subglobose, slightly raised blistering areas, central ostiole with periphyses
Peridium	Textura angularis	Textura angularis	Textura angularis
Hamathecium	Hyaline, septate paraphyses, longer than asci	Hyaline, septate paraphyses, longer than asci	Hyaline, septate paraphyses, longer than asci
Asci	Cylindrical, unitunicate, a small non-amyloid apical ring	Long cylindrical, pedicellate, unitunicate, an oblong to wedge-shaped, refractive, apical ring and some with a refractive circular body below	Cylindrical to cylindric-clavate, unitunicate, pedicellate, doughnut-shaped, refractive, J- apical ring
Ascospores	Filiform, aseptate, hyaline or pale-yellowish in mass, parallel or spiral, with appendage and refringent septum-like bands or absent	Filiform, aseptate, hyaline or pale-yellowish in mass, parallel or spiral, with apical appendages and refringent bands or absent	Clavate, thin pedicellate, aseptate, hyaline, parallel, no appendage and refringent band
Asexual morph	Only found in *L. appendiculatum* and *L. elaeidis*, conidiophore arising from the aerial mycelium, conidiogenous cells phialidic, smooth, translucent brown, conidia clavate to fusiform, straight or slightly curved or slightly sinuous, unicellular, smooth, colourless	Undetermined	Undetermined
Others	Colonies on MEA and PDA growing slowly	Colonies on MEA growing slowly. Ascospores on MEA produced appressoria-like structures at each tip of germ tube, only found in *N. rachidis*	Colonies on PDA grow faster
References	Hyde (1992b), Konta et al. (2017), Thongkantha et al. (2003)	Hyde et al. (2019), Senwanna et al. (2018), Hyde et al. (1998)	This study

### Claviformispora phyllostachydis

Taxon classificationFungiChaetosphaerialesLinocarpaceae

X. L. Xu & C. L. Yang sp. nov.

CA96D7CB-79F6-5E16-B290-C135E6A2D227

Index Fungorum identifier: IF557396

Fig. 2

#### Type.

China, Sichuan Province, Ya’an City, Yucheng Distinct, Kongping Township, alt. 1133 m, 29°50.14'N, 103°03'E, on dead branches of *Phyllostachys heteroclada* Oliv. (Poaceae), 11 December 2016, C. L. Yang and X. L. Xu, YCL201612002 (SICAU 16-0007, ***holotype***; MFLU 18-1217, ***isotype***), ex-type living culture, SICAUCC 16-0004 = MFLUCC 18-1230.

**Figure 2.**
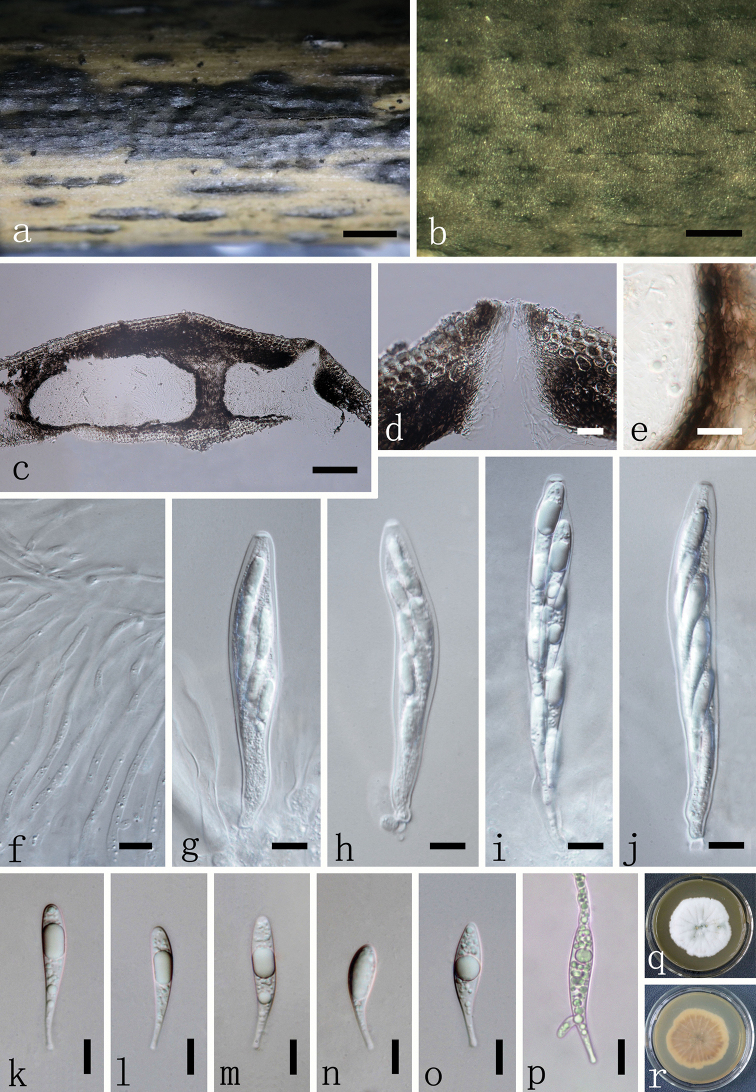
*Claviformispora phyllostachydis* (SICAU 16-0007, holotype) **a, b***Stromata* on host substrate **c** section through ascoma with ascomata **d** ostiole with periphyses **e** peridium **f** paraphyses **g–j** asci **k–o** ascospores **p** germinated ascospore **q, r** colony on PDA after 7 days. Scale bars: 2 mm (**a**), 500 μm (**b**), 100 μm (**c**), 20 μm (**d, e**), 10 μm (**f–p**).

#### Etymology.

The specific epithet refers to the host genus *Phyllostachys*.

#### Description.

*Saprobic* on dead branches of *Phyllostachys heteroclada* Oliv. ***Sexual morph***: *Stromata* solitary comprising elliptical areas or aggregated in large black areas, slightly raised with slit-like openings presenting on host surface. *Ascomata* 120–240 μm high × 220–490 μm diameter (x̄ = 189 × 345 μm, n = 20), perithecial, immersed, central, papillate ostiole with periphyses, oval-globose in section, the cells between the perithecia are composed with tissue of stromata and host. *Peridium* 20–40 μm wide (x̄ = 33 μm, n = 10), outer cells merging with the host tissues, composed of pale to dark brown cells of *textura angularis*. *Hamathecium* comprising hyaline, hypha-like, septate paraphyses, occasionally branched, longer than asci, wider at the base, 2–4 μm diameter (x̄ = 2.7 μm, n = 20) tapering towards the apex, 0.78–1.20 μm diameter (x̄ = 0.98 μm, n = 20). *Asci* 90–160 × 9–15 μm (x̄ = 118 × 13 μm, n = 20), 8-spored, cylindrical to cylindric-clavate, unitunicate, short pedicellate, apically rounded, with a massive, doughnut-shaped, refractive, J- reaction, apical ring. *Ascospores* 35–50 × 5.7–8.6 μm (x̄ = 45.7 × 7.0 μm, n = 40), overlapping uniseriate or 2-seriate, claviform typically with a thin pedicel, aseptate, hyaline, straight or slight curved, without appendage and septum-like bands, guttulate when maturity. ***Asexual morph***: Undetermined.

#### Culture characters.

Ascospores germinated on PDA within 12 hours at both ends. Colonies on PDA reaching 5 cm diameter after 7 days at 25 °C, white to grey with strong radiations outwards on forward side. Colonies became dark brown and black on the reverse after a long time of cultivation. The hyphae are septate, branched, smooth.

## Discussion

This study establishes a new genus and also provides further insights into the phylogeny of members associated with Linocarpaceae. Morphologically-based examinations of *Claviformispora* (as discussed above) clearly show that the morphological circumscriptions (familial concept) of species should be broadened and possibly indicate that this family is much more diverse than expected. Our collection can be clearly distinguished from other groups of similar fungi in Linocarpaceae with its interesting ascospore morphology. In addition, we also noted some peculiarities in the DNA sequences we analysed. A comparison of ITS sequences, based on BLAST reveals 34%, 26% and 30% base pair differences with *L. cocois* (MFLUCC 15-0812), *N. arengae* (MFLUCC 15-0323) and *N. rachidis* (MFLUCC 15-0814a), respectively. There are more than 9% and 5% sequence differences with the three taxa when the LSU and SSU rDNA sequences were compared respectively. Following the guidelines recommended by Jeewon and Hyde (2016), there are therefore sufficient grounds to establish a new species at the genus rank.

Species of Linocarpaceae have been found on Arecaceae, Poaceae, Euphorbiaceae, Zingiberaceae, Pandanaceae, Fagaceae, Fabaceae and Smilacaceae, including *Arenga*, *Attalea*, *Calamus*, *Trachycarpus*, *Acrocomia*, *Archontophoenix*, *Cocos*, *Daemonorops*, *Licuala*, *Livistona*, *Plectocomia*, *Phoenix*, *Raphia*, *Sabal*, *Mauritia*, *Nypa*, *Elaeis*, *Pinanga*, *Eugeissona*, *Pennisetum*, *Gramineae*, *Stipa*, unidentified bamboo, *Hevea*, *Manihot*, *Alpinia*, *Pandanus*, *Quenrcus*, *Cajanus* and *Smilax* (Hyde 1988, 1992a, b; Dulymamode et al. 1998; Hyde et al. 1998; Hyde and Alias 1999; Thongkantha et al. 2003; Cai et al. 2004; Bhilabutra et al. 2006; Vitoria et al. 2013; Konta et al. 2017; Senwanna et al. 2018). More than 50% of the species were recorded from hosts of the Arecaceae. Species in Linocarpaceae are mostly saprobic, except *Linocarpon palmetto* which was discovered as a pathogen of *Sabal palmetto* in Florida (Barr 1978). Four species in Linocarpaceae from Poaceae have been reported so far, including *Neolinocarpon penniseti* on *Pennisetum purpureum* (Bhilabutra et al. 2006), *Linocarpon williamsii* on *Gramineae* sp. (Hansford 1954), *L. stipae* on *Stipa* sp. (Hansford 1954) and *L. bambusicola* on unidentified bamboo submerged in a river (Cai et al. 2004).

*Phyllostachys heteroclada*, mainly a food source and use as a material in the weaving industry, is distributed along the Yellow River Valley and the southern Provinces in China. It is common in the mountainous areas of Sichuan Province with distribution up to 1,500 m above sea level (Yi 1997; Yi et al. 2008). There is a large area of pure forest in Yibin, Leshan and Ya’an Cities and sporadic distribution in other areas. According to preliminary statistics, bambusicolous fungi from seven orders (excluding fungi referred to as Sordariomycetes*incertae sedis*) have been recorded on *P. heteroclada*, including Hypocreales, Ostropales, Pleosporales, Phyllachorales, Pucciniales, Ustilaginales and Xylariales, of which Pleosporales is the largest one. Most bambusicolous fungi in China were recorded with inadequate morphological descriptions or molecular data. The early known fungi on *P. heteroclada* are documented as *Aciculosporium take*, *Ellisembia pseudoseptata*, *Fusarium oxysporum*, *F. semitectum*, *Phyllachora gracilis*, *Ph. orbicular*, *Shiraia bambusicola*, *Stereostratum corticioides* and *Ustilago shiraiana* (Zhou et al. 2001; Xu et al. 2006). In recent years, some new records and taxa, viz. *Bambusicola subthailandica*, *B. sichuanensis*, *Neostagonosporella sichuanensis*, *Parakarstenia phyllostachydis*, *Phyllachora heterocladae*, *Podonectria sichuanensis*, *Arthrinium yunnanum* and *A. phyllostachium* have been reported (Yang et al. 2019a, b, c, d, e, f). Here, we introduce a new genus in order Chaetosphaeriales, which is a contribution to fungal diversity on *P. heteroclada*.

## Supplementary Material

XML Treatment for Linocarpaceae

XML Treatment for Claviformispora

XML Treatment for Claviformispora phyllostachydis

## Supplementary materials

Supplementary material 1 Figure S1 Data type multimedia https://binary.pensoft.net/file/432595This dataset is made available under the Open Database License (http://opendatacommons.org/licenses/odbl/1.0/). The Open Database License (ODbL) is a license agreement intended to allow users to freely share, modify, and use this Dataset while maintaining this same freedom for others, provided that the original source and author(s) are credited.Xiu-Lan Xu, Chun-Lin Yang, Rajesh Jeewon, Dhanushka N. Wanasinghe, Ying-Gao Liu, Qian-Gang Xiao

Supplementary material 2 Figure S2 Data type multimedia https://binary.pensoft.net/file/432596This dataset is made available under the Open Database License (http://opendatacommons.org/licenses/odbl/1.0/). The Open Database License (ODbL) is a license agreement intended to allow users to freely share, modify, and use this Dataset while maintaining this same freedom for others, provided that the original source and author(s) are credited.Xiu-Lan Xu, Chun-Lin Yang, Rajesh Jeewon, Dhanushka N. Wanasinghe, Ying-Gao Liu, Qian-Gang Xiao

## References

[B1] Bahl J (2006) Molecular evolution of three morphologically similar families in the Xylariomycetidae (Apiosporaceae, Clypeosphaeriaceae, Hyponectriaceae). PhD Thesis, The University of Hong Kong, China.

[B2] Barr ME (1978) The Diaporthales in North America: with emphasis on *Gnomonia* and its segregates. Mycologia Memoir 7: 1–232.

[B3] Bhilabutra W Lumyong S Jeewon R McKenzie EHC Hyde KD (2006) *Neolinocarpon penniseti* sp. nov. on the grass *Pennisetum purpureum* (Poaceae). Cryptogamie, Mycologie 27(4): 305–310.

[B4] Cai L Zhang KQ McKenzie EHC Hyde KD (2004) *Linocarpon bambusicola* sp. nov. and *Dictyochaeta curvispora* sp. nov. from bamboo submerged in freshwater. Nova Hedwigia 78(3): 439–445. 10.1127/0029-5035/2004/0078-0439

[B5] Chomnunti P Hongsanan S Hudson BA Tian Q Peršoh D Dhami MK Alias AS Xu J Liu X Stadler M Hyde KD (2014) The sooty moulds. Fungal Diversity 66(1): 1–36. 10.1007/s13225-014-0278-5

[B6] Dai DQ Phookamsak R Wijayawardene NN Li WJ Bhat DJ Xu JC Tayor JE Hyde KD Chukeatirote K (2017) Bambusicolous fungi. Fungal Diversity 82(1): 1–105. 10.1007/s13225-016-0367-8

[B7] Dulymamode R Cannon PF Peerally A (1998) Fungi from Mauritius: *Linocarpon* species on *Pandanus*. Mycological Research 102(11): 1331–1337. 10.1017/S0953756298006406

[B8] Hall TA (1999) BioEdit: a user-friendly biological sequence alignment editor and analysis program for Windows 95/98/NT. Nucleic Acids Symposium Series 41: 95–98.

[B9] Hansford CG (1954) Australian Fungi. II. New species and revisions. Proceedings of the Linnean Society of New South Wales 79: 97–141.

[B10] Hongsanan S Maharachchikumbura SSN Hyde KD Samarakoon MC Jeewon R Zhao Q Al-sadi AM Bahkali AH (2017) An updated phylogeny of Sordariomycetes based on phylogenetic and molecular clock evidence. Fungal Diversity 84(1): 25–41. 10.1007/s13225-017-0384-2

[B11] Huhndorf SM Miller AN (2011) A molecular re-appraisal of taxa in the *Sordariomycetidae* and a new species of *Rimaconus* from New Zealand. Studies in Mycology 68: 203–210. 10.3114/sim.2011.68.09 21523195 PMC3065991

[B12] Huhndorf SM Miller AN Fernández FA (2004) Molecular systematics of the Sordariales: the order and the family Lasiosphaeriaceae redefined. Mycologia 96(2): 368–387. 10.1080/15572536.2005.11832982 21148859

[B13] Hyde KD (1992a) Fungi from decaying intertidal fronds of *Nypa fruticans*, including three new genera four new species. Botanical Journal of the Linnean Society 110(2): 95–110. 10.1111/j.1095-8339.1992.tb00284.x

[B14] Hyde KD (1992b) Fungi from palms. I. The genus *Linocarpon*, a revision. Sydowia 44: 32–54.

[B15] Hyde KD (1997) Additions to the genus *Linocarpon* (Ascomycetes: Hyponectriaceae). Botanical Journal of the Linnean Society 123(2): 109–131. 10.1111/j.1095-8339.1997.tb01407.x

[B16] Hyde KD (1988) The genus *Linocarpon* from the mangrove palm *Nypa fruticans*. Transactions of the Mycological Society of Japan 29: 339–350.

[B17] Hyde KD Alias SA (1999) *Linocarpon angustatum* sp. nov., and *Neolinocarpon nypicola* sp. nov. from petioles of *Nypa fruticans*, and a list of fungi from aerial parts of this host. Mycoscience 40(2): 145–149. 10.1007/BF02464293

[B18] Hyde KD Norphanphoun C Maharachchikumbura SSN Bhat DJ Jones EBG Bundhun D Chen YJ Bao DF Boonmee S Calabon MS Chaiwan N Chethana KWT Dai DQ Dayarathne MC Devadatha B Dissanayake AJ Dissanayake LS Doilom M Dong W Fan XL Goonaselara ID Hongsanan S Huang SK Jayawardena RS Jeewon R Karunarathna A Konta S Kumar V Lin CG Liu JK Liu NG Luangsa-ard J Lumyong S Luo ZL Marasinghe DS McMenzie EHC Niego AGT Niranjan M Perera RH Phukhamsakda C Rathnayaka AR Samarakoon MC Samarakoon SMBC Sarma VV Senanayake IC Shang QJ Stadler M Tibpromma S Wanasinghe DN Wei DP Wijayawardene NN Xiao YP Yang J Zeng XY Zhang SN Xiang MM (2020) Refined families of Sordariomycetes. Mycosphere 11(1): 305–1059. 10.5943/mycosphere/11/1/7

[B19] Hyde KD Taylor JE Fröhlich J (1998) Fungi from palms. XXXIV. The genus *Neolinocarpon* with five new species and one new combination. Fungal Diversity 1: 115–131.

[B20] Hyde KD Tennakoon DS Jeewon R Bhat DJ Maharachchikumbura SSN Rossi W Leonardi M Lee HB Mum HY Houbraken J Nguyen TTT Jeon SJ Frisvad JC Wanasinghe DN Lücking R Aptroot A Cáceres MES Karunarathna SC Hongsanan S Phookamsak R de Silva NI Thambugala KM Jayawardena RS Senanayake IC Boonmee S Chen J Luo ZL Phukhamsakda C Pereira OL Abreu VP Rosado AWC Bart B Randrianiohany E Hofstetter V Gibertoni TB da Silva Soares AM Plautz Jr HL Sotão HMP Xavier WKS Bezerra JDP de Oliveira TGL de Souza-Motta CM Magalhães OMC Bundhun D Harishchandra D Manawasinghe IS Dong W Zhang SN Bao DF Samarakoon MC Pem D Karunarathna A Lin CG Yang J Perera RH Kumar V Huang SK Dayarathne MC Ekanayaka AH Jayasiri SC Xiao YP Konta S Niskanen T Liimatainen K Dai YC Ji XH Tian XM Mešić A Singh SK Phutthacharoen K Cai L Sorvongxay T Thiyagaraja V Norphanphoun C Chaiwan N Lu YZ Jiang HB Zhang JF Abeywickrama PD Aluthmuhandiram JVS Brahmanage RS Zeng M Chethana T Wei DP Réblová M Fournier J Nekvindová J do Nascimento Barbosa R dos Santos JEF de Oliveira NT Li GJ Ertz D Shang QJ Phillips AJL Kuo CH Camporesi E Bulgakov TS Lumyong S Jones EBG Chomnunti P Gentekaki E Bungartz F Zeng XY Fryar S Tkalčec Z Liang JM Li GS Wen TC Singh PN Gafforov Y Promputtha I Yasanthika E Goonasekara ID Zhao RL Zhao Q Kirk PM Liu JK Yan JY Mortimer PE Xu JC Doilom M (2019) Fungal diversity notes 1036–1150: taxonomic and phylogenetic contributions on genera and species of fungal taxa. Fungal Diversity 96: 1–242. 10.1007/s13225-019-00429-2

[B21] Index Fungorum (2020) Index Fungorum. http://www.indexfungorum.org/Names/Names.asp [Accessed 4 May 2020]

[B22] Jayasiri SC Hyde KD Ariyawansa HA Bhat J Buyck B Cai L Dai YC Abd-Elsalam KA Ertz D Hidayat I Jeewon R Jones EBG Bahkali AH Karunarathna SC Liu JK Luangsa-ard JJ Lumbsch HT Maharachchikumbura SSN McKenzie EHC Moncalvo JM Ghobad-Nejhad M Nilsson H Pang KL Pereira O Phillips AJL Raspé O Rollins AW Romero AI Etayo J Selçuk F Stephenson SL Suetrong S Taylor JE Tsui CKM Vizzini A Abdel-Wahab MA Wen TC Boonmee S Dai DQ Daranagama DA Dissanayake AJ Ekanayaka AH Fryar SC Hongsanan S Jayawardena RS Li WJ Perera RH Phookamsak R de Silva NI Thambugala KM Tian Q Wijayawardene NN Zhao RL Zhao Q Kang JC Promputtha I (2015) The Faces of Fungi database: fungal names linked with morphology, phylogeny and human impacts. Fungal Diversity 74(1): 3–18. 10.1007/s13225-015-0351-8

[B23] Jeewon R Hyde KD (2016) Establishing species boundaries and new taxa among fungi: recommendations to resolve taxonomic ambiguities. Mycosphere 7(11): 1669–1677. 10.5943/mycosphere/7/11/4

[B24] Jeewon R Liew ECY Hyde KD (2003) Molecular systematics of the *Amphisphaeriaceae* based on cladistic analyses of partial LSU rDNA gene sequences. Mycological Research 107(12): 1392–1402. 10.1017/S095375620300875X 15000240

[B25] Jeewon R Liew ECY Simpson JA Hodgkiss IJ Hyde KD (2003) Phylogenetic significance of morphological characters in the taxonomy of *Pestalotiopsis* species. Molecular Phylogenetics and Evolution 27(3): 372–383. 10.1016/S1055-7903(03)00010-1 12742743

[B26] Katoh K Rozewicki J Yamada KD (2019) MAFFT online service: multiple sequence alignment, interactive sequence choice and visualization. Briefings in Bioinformatics 20: 1160–1166. 10.1093/bib/bbx108 28968734 PMC6781576

[B27] Konta S Hongsanan S Liu JK Eungwanichayapant PD Jeewon R Hyde KD Maharachchikumbura SSN Boonmee S (2017) *Leptosporella* (*Leptosporellaceae* fam. nov.) and *Linocarpon* and *Neolinocarpon* (*Linocarpaceae* fam. nov.) are accommodated in Chaetosphaeriales. Mycosphere 8(10): 1943–1974. 10.5943/mycosphere/8/10/16

[B28] Lin CG McKenzie EHC Liu JK Jones EBG Hyde KD (2019) Hyaline-spored chaetosphaeriaceous hyphomycetes from Thailand and China, with a review of the family Chaetosphaeriaceae. Mycosphere 10(1): 655–700. 10.5943/mycosphere/10/1/14

[B29] Lu YZ Liu KJ Hyde KD Bhat DJ Xiao YP Tian Q Wen TC Boonmee S Kang JC (2016) *Brunneodinemasporium jonesii* and *Tainosphaeria jonesii* spp. nov. (Chaetosphaeriaceae, Chaetosphaeriales) from southern China. Mycosphere 7(9): 1322–1331. 10.5943/mycosphere/7/9/6

[B30] Lumbsch HT Huhndorf SM (2010) Myconet (Vol. 14). Part One. Outline of Ascomycota–2009. Part Two. Notes on Ascomycete Systematics. Nos. 4751–5113. Fieldina Life and Earth Sciences 1: 1–64. 10.3158/1557.1

[B31] Maddison W P Maddison DR (2019) Mesquite: a modular system for evolutionary analysis. Version 3.61. http://www.mesquiteproject.org

[B32] Miller MA Pfeiffer WT Schwartz T (2010) Creating the CIPRES Science Gateway for Inference of Large Phylogenetic Tree. Gateway Computing Environments Workshop (GCE), 8 pp. 10.1109/GCE.2010.5676129

[B33] Nylander JAA MrModeltest v2 (2004) Program distributed by the author. https://www.researchgate.net/publication/285805344

[B34] Penzig AJO Saccardo PA (1897) Diagnoses fungorum novorum in Insula Java collectorum. Series. I. Malpighia 11: 387–409. 10.5962/bhl.title.4921

[B35] Poonyth AD Hyde KD Wong SW Peerally A (2000) Ultrastructure of asci and ascospore appendages in *Linocarpon appendiculatum* and *L. nypae*. Botanica Marina 43(3): 213–221. 10.1515/BOT.2000.023

[B36] Rambaut A Drummond A (2016) FigTree: Tree figure drawing tool, version 1.4.3. http://tree.bio.ed.ac.uk/software/figtree/ [accessed 04 May 2020]

[B37] Rehner S (2001) Primers for elongation factor 1-α (EF1-α). http://ocid.NACSE.ORG/research/deephyphae/EF1primer.pdf [accessed 04 May 2020]

[B38] Senwanna C Phookamsak R Bahkali AH Elgorban AM Cheewangkoon R Hyde KD (2018) *Neolinocarpon phayaoense* sp. nov. (Linocarpaceae) from Thailand. Phytotaxa 362(1): 077–086. 10.11646/phytotaxa.362.1.6

[B39] Stamatakis A (2014) RAxML version 8: a tool for phylogenetic analysis and post-analysis of large phylogenies. Bioinformatics 30(9): 1312–1313. 10.1093/bioinformatics/btu033 24451623 PMC3998144

[B40] Sydow H Sydow P (1917) Beitrag zur Kenntniss der Pilzflora der Philippinen-Inseln. Annales Mycologici 15: 165–268.

[B41] Thongkantha S Lumyong S Lumyong P Whitton SR McKenzie EHC Hyde KD (2003) Microfungi on the Pandanaceae: *Linocarpon lammiae* sp. nov., *L. siamensis* sp. nov. and *L. suthepensis* sp. nov. are described with a key to *Linocarpon* species from the Pandanaceae. Mycologia 95(2): 360–367. 10.1080/15572536.2004.11833122 21156623

[B42] Vilgalys R Hester M (1990) Rapid genetic identification and mapping of enzymatically amplified ribosomal DNA from several *Cryptococcus* species. Journal of Bacteriology 172(8): 4238–4246. 10.1128/JB.172.8.4238-4246.1990 2376561 PMC213247

[B43] Vitoria NS Cavalcanti MAQ Santos CDD Pereira J Bezerra JL (2013) *Neolinocarpon attaleae* sp nov on *Attalea funifera* (Arecaceae) from Brazil. Mycotaxon 123(1): 141–145. 10.5248/123.141

[B44] Wei MJ Zhang H Dong W Boonmee S Zhang D (2018) Introducing *Dictyochaeta qauatica* sp. nov. and two new species of *Chloridium* (Chaetosphaeriaceae, Sordariomycetes) from aquatic habitat. Phytotaxa 362(2): 187–199. 10.11646/phytotaxa.362.2.5

[B45] White TJ Bruns T Lee S Taylor J (1990) Amplifcation and direct sequencing of fungal ribosomal RNA genes for phylogenetics. PCR Protocols: A Guide to Methods and Applications 18: 315–322. 10.1016/B978-0-12-372180-8.50042-1

[B46] Wijayawardene N Hyde KD Al-Ani LKT Tedersoo L Haelewaters D Rajeshkumar KC Zhao RL Aptroot A Leontyev DV Saxena RK Tokarev YS Dai DQ Letcher PM Stephenson SL Ertz D Lumbsch HT Kukwa M Issi IV Madrid H Phillips AJL Selbmann L Pfliegler WP Horváth E Bensch K Kirk P Kolaříková Z Raja HA Radek R Papp V Dima B Ma J Malosso E Takamatsu S Rambold G Gannibal PB Triebel D Gautam AK Avasthi S Suetrong S Timdal E Fryar SC Delgado G Réblová M Doilom M Dolatabadi S Pawłowska J Humber RA Kodsueb R Sánchez-Castro I Goto BT Silva DKA de Souza FA Oehl F da Silva GA Silva IR Błaszkowski J Jobim K Maia LC Barbosa FR Fiuza PO Divakar PK Shenov BD Castañeda-Ruiz RF Somrithipol S Karunarathna SC Tibpromma S Mortimer PE Wanasinghe DN Phookamsak R Xu JC Wang Y Tian FH Alvarado P Li DW Kušan I Matočec N Maharachchikumbura SSN Papizadeh M Heredia G Wartchow F Bakhshi M Boehm E Youssef N Hustad VP Lawrey JD de A Santiago ALCM Tian Q Houbraken J Hongsanan S Tanaka K Dissanayake AJ Monteiro JS Grossart HP Suija A Weerakoon G Etayo J Tsurykau A Kuhnert E Vázquez V Mungai P Damm U Li QR Zhang H Boonmee S Lu YZ Becerra AG Kendrick B Brearley FQ Motiejūnaitė J Sharma B Khare R Gaikwad S Wijesundara DSA Tang LZ He MQ Flakus A Rodriguez-Flakus P Zhurbenko MP McKenzie EHC Stadler M Bhat DJ Liu JK Raza M Jeewon R Nassonova ES Prieto M Ja yalal RGU Yurkov A Schnittler M Shchepin ON Novozhilov YK Liu P Cavender JC Kang YQ Mohammad S Zhang LF Xu RF Li YM Dayarathne MC Ekanayaka AH Wen TC Deng CY Lateef AA Pereira OL Navathe S Hawksworth DL Fan XL Dissanayake LS Erdoğdu M (2020) Outline of Fungi and fungi-like taxa. Mycosphere 11(1): 1060–1456. 10.5943/mycosphere/11/1/8

[B47] Xu MQ Dai YC Fan SH Jin LX Lv Q Tian GZ Wang LF (2006) Records of bamboo diseases and the taxonomy of their pathogens in China (I). Forest Research 19(6): 692–699. 10.13275/j.cnki.lykxyj.2006.06.004

[B48] Yang CL Baral HO Xu XL Liu YG (2019a) *Parakarstenia phyllostachydis*, a new genus and species of non-lichenized Odontotremataceae (Ostropales, Ascomycota). Mycological Progress 18(6): 833–845. 10.1007/s11557-019-01492-4

[B49] Yang CL Xu XL Dong W Wanasinghe DN Liu YG Hyde KD (2019b) Introducing *Arthrinium phyllostachium* sp. nov. (Apiosporaceae, Xylariales) on *Phyllostachys heteroclada* from Sichuan Province, China. Phytotaxa 406(2): 91–110. 10.11646/phytotaxa.406.2.2

[B50] Yang CL Xu XL Liu YG (2019c) Two new species of *Bambusicola* (Bambusicolaceae, Pleosporales) on *Phyllostachys heteroclada* from Sichuan, China. Nova Hedwigia 108(3): 527–545. 10.1127/nova_hedwigia/2019/0526

[B51] Yang CL Xu XL Liu YG Hyde KD McKenzie EHC (2019d) A new species of *Phyllachora* (Phyllachoraceae, Phyllachorales) on *Phyllostachys heteroclada* from Sichuan, China. Phytotaxa 392(3): 186–196. 10.11646/phytotaxa.392.3.2

[B52] Yang CL Xu XL Liu YG (2019e) *Podonectria sichuanensis*, a potentially mycopathogenic fungus from Sichuan Province in China. Phytotaxa 402(5): 219–231. 10.11646/phytotaxa.402.5.1

[B53] Yang CL Xu XL Wanasinghe DN Jeewon R Phookamsak R Liu YG Liu LJ Hyde KD (2019f) *Neostagonosporella sichuanensis* gen. et sp. nov. (Phaeosphaeriaceae, Pleosporales) on *Phyllostachys heteroclada* (Poaceae) from Sichuan Province, China. MycoKeys 46: 119–150. 10.3897/mycokeys.46.32458 PMC6389646 30814907

[B54] Yi TP (1997) Bamboos Flora of Sichuan. China Forestry Publishing House, 358 pp.

[B55] Yi TP Shi JY Ma LS Wang HT Yang L (2008) Iconographia Bambusoidearum Sinicarum. Science Press, 766 pp.

[B56] Zhou DQ Hyde KD Wu XL (2001) New records of *Ellisembia*, *Penzigomyces*, *Sporidesmium* and *Repetophragma* species on Bamboo from China. Acta Botanica Yunnanica 23(1): 45–51.

