# Supplementary figures and images for: Morpho-molecular diversity of Linocarpaceae (Chaetosphaeriales): Claviformispora gen. nov. from decaying branches of Phyllostachys heteroclada
Source: MycoKeys. 2020 Jul 16;70:1–17. doi: 10.3897/mycokeys.70.54231 (PMC7381431; doi:10.3897/mycokeys.70.54231)

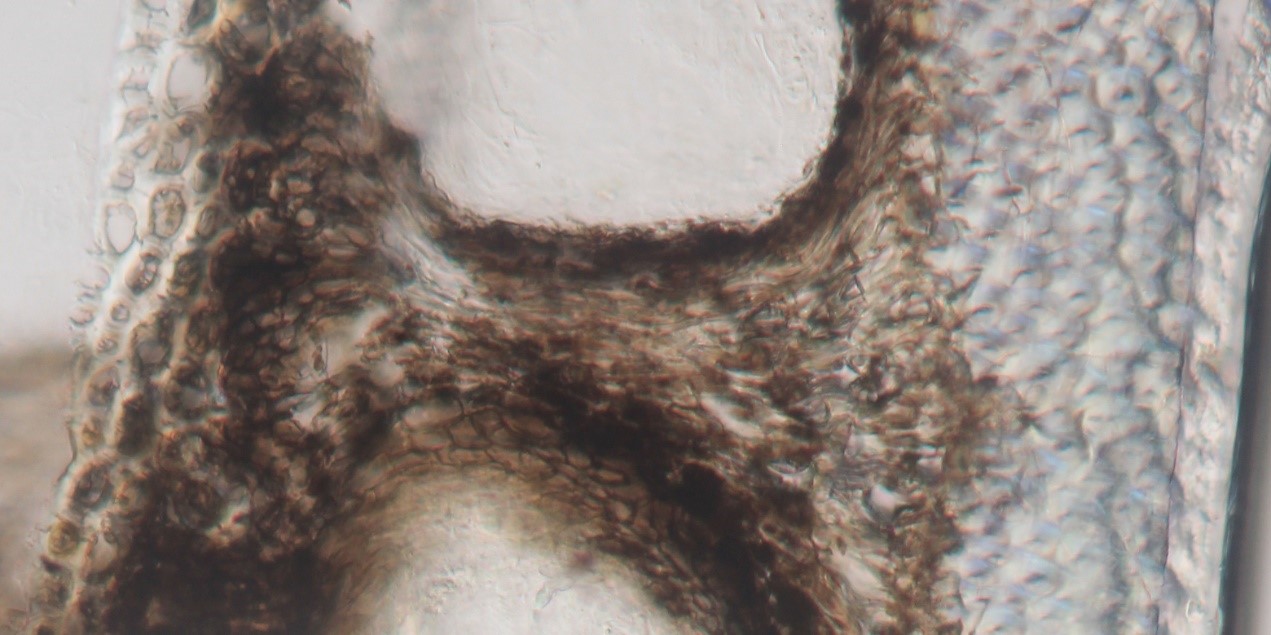

Supplement: Supplementary material 1 — Figure S1 [file mycokeys-70-001-s001.jpg]

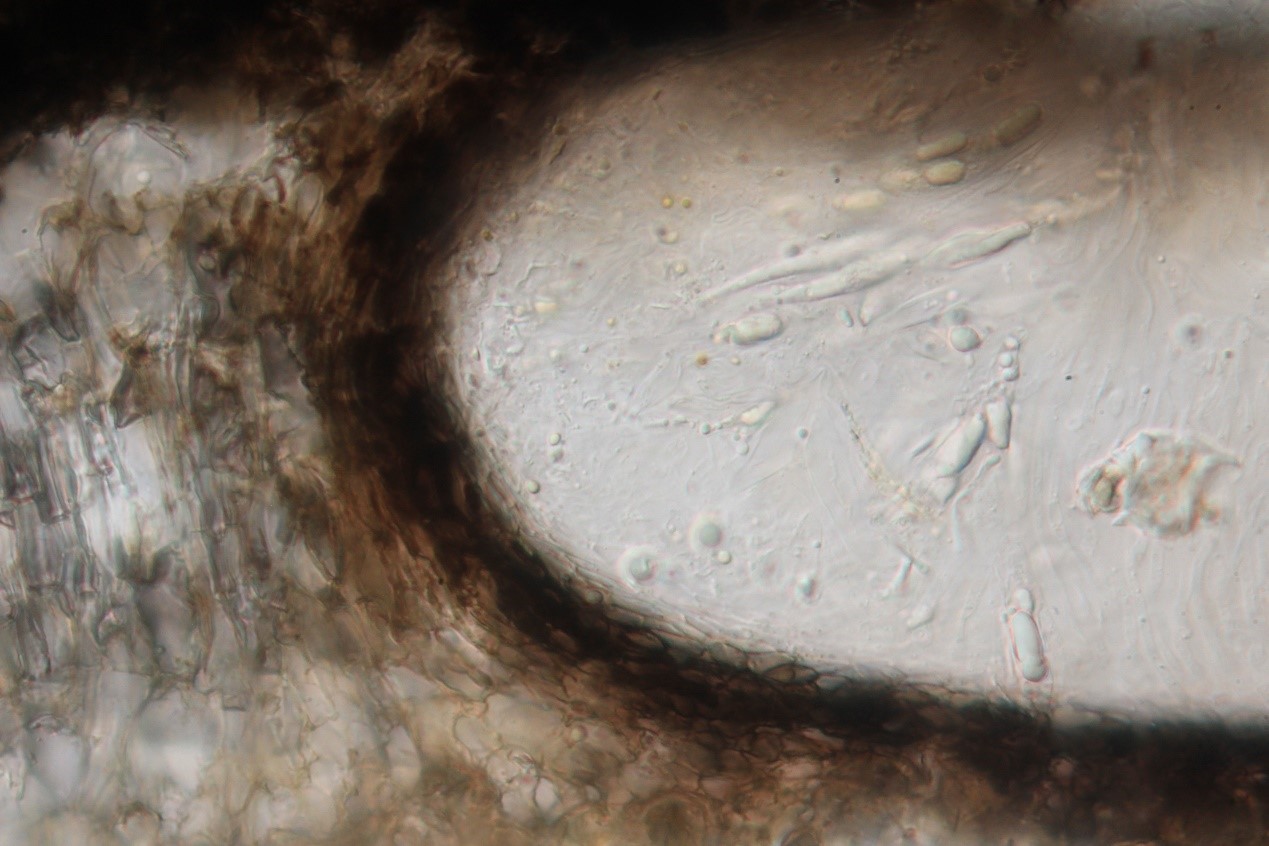

Supplement: Supplementary material 2 — Figure S2 [file mycokeys-70-001-s002.jpg]
